# Predicting tomato water consumption in a hydroponic greenhouse: contribution of light interception models

**DOI:** 10.3389/fpls.2023.1264915

**Published:** 2023-11-28

**Authors:** Konstantinos Florakis, Samis Trevezas, Véronique Letort

**Affiliations:** ^1^ Department of Mathematics, National and Kapodistrian University of Athens, Athens, Greece; ^2^ Université Paris-Saclay, CentraleSupélec, Mathématiques et Informatique pour la Complexité et les Systèmes, Gif-sur-Yvette, France

**Keywords:** GreenLab model, greenhouse, MLE, Stochastic Segmentation of input Energy, water consumption, Beer-Lambert law, light interception

## Abstract

In recent years, hydroponic greenhouse cultivation has gained increasing popularity: the combination of hydroponics’ highly efficient use of resources with a controlled environment and an extended growing season provided by greenhouses allows for optimized, year-round plant growth. In this direction, precise and effective irrigation management is critical for achieving optimal crop yield while ensuring an economical use of water resources. This study explores techniques for explaining and predicting daily water consumption by utilizing only easily readily available meteorological data and the progressively growing records of the water consumption dataset. In situations where the dataset is limited in size, the conventional purely data-based approaches that rely on statistically benchmarking time series models tend to be too uncertain. Therefore, the objective of this study is to explore the potential contribution of crop models’ main concepts in constructing more robust models, even when plant measurements are not available. Two strategies were developed for this purpose. The first strategy utilized the Greenlab model, employing reference parameter values from previously published papers and re-estimating, for identifiability reasons, only a limited number of parameters. The second strategy adopted key principles from crop growth models to propose a novel modeling approach, which involved deriving a Stochastic Segmentation of input Energy (SSiE) potentially absorbed by the elementary photosynthetically active parts of the plant. Several model versions were proposed and adjusted using the maximum likelihood method. We present a proof-of-concept of our methodology applied to the ekstasis Tomato, with one recorded time series of daily water uptake. This method provides an estimate of the plant’s dynamic pattern of light interception, which can then be applied for the prediction of water consumption. The results indicate that the SSiE models could become valuable tools for extracting crop information efficiently from routine greenhouse measurements with further development and testing. This, in turn, could aid in achieving more precise irrigation management.

## 1 Introduction

Recent years have witnessed considerable research interest in the topic of water uptake by plants, particularly in the context of hydroponic greenhouse cultivation. For tomato plants in particular, numerous studies have stressed the importance of water uptake in comparing different cultivation methodologies. For instance, Reina-Sánchez et al. (2005) explored the effects of salinity concentration on water uptake, and Biswas et al. (2016) evaluated the impact of differing drip irrigation techniques. Moreover, investigations have been carried out to assess the influence of nitrogen supply on growth, yield, and Water Use Efficiency (WUE), incorporating biomass measurements either in field conditions (Cheng et al., 2021) or within a hydroponic greenhouse environment (Martínez-Ruiz et al., 2019). In Sigrimis et al. (2001), a distinct approach has been proposed, which suggests a methodology predicated upon measurements taken postirrigation, accompanied by online re-estimation of the parameters of their model. While the study reported a high degree of accuracy for the proposed methodology, it is crucial to highlight its significant dependence on measurements taken after each individual irrigation event, requiring sophisticated instruments. This continuous monitoring may challenge the average producer, who may lack access to such advanced tools. Furthermore, the solution appears to be largely engineering-based, neglecting the biological representation of the plant’s development. Adhering to a common practice of daily measurements, the challenge in our setting consists in making the best use of limited information from data that concerns only water uptake and easily accessible meteorological data/parameters. This framework resembles the typical evaluation scheme that an average producer might employ to assess the productivity of their cultivation (Resh, 2022). However, despite the potential benefits for average producers, the proposed simplified experimental protocol may face serious limitations if data are not sufficiently informative due to measurement or even modeling errors caused by oversimplified assumptions. These limitations will be further discussed in the sequel.

Plant water uptake is directly related to many greenhouse functions such as electric power usage (fertilizer mixer, climatic regulating facilities, etc.), fertilizer consumption, and yield production and quality (Resh, 2022). Predicting water consumption in a given day could help the average producer regulate these costs, prevent excess-deficit irrigation, and increase production. Another aspect of the problem is water waste: In an extensive study spanning 165 countries, the Food and Agriculture Organization of the United Nations (FAO) estimated the total requirements and measurements of total withdrawals per country, thus documenting a 56% irrigation efficiency only (Food and Agriculture Organization of the United Nations (FAO), 2012).

Water consumption prediction can primarily be accomplished through two distinct methods. The first approach is purely statistical, relying entirely on analyzing data series (Sigrimis et al., 2001). The second approach employs process-based models or Functional-Structural Plant Models (FSPMs) (Sievänen et al., 2014), which, despite requiring detailed and potentially costly longitudinal plant measurements, are invaluable for their ability to convey information about underlying physiological processes and potential interactions. The GreenLab model (Yan et al., 2004) is such an FSPM, combining both functional and structural description of metabolic processes with phytomer-level structures (De Reffye et al., 2021), and integrating the effects of water dynamics on plant growth (Wang et al., 2012), thus allowing optimization of water supply (Wu et al., 2005). However, in many cases of professional practices where access to detailed plant measurements is unavailable, neither of these approaches directly applies. Our objective, therefore, is to find the appropriate level of complexity for a model that remains as mechanistic as possible. The necessity of maintaining a mechanistic orientation stems from a few key reasons: the interpretability of certain parameters allows using *a priori* biological knowledge and, crucially, our data’s limited and uncertain nature. This uncertainty makes a purely statistical approach infeasible, underscoring the need for a method that captures the underlying physiological processes to a suitable extent. To this end, we developed a new set of aggregated model versions, which inherit from the GreenLab principles but differ in the representation of the Light Interception Ratio (LIR) of the plant.

In GreenLab, as in most process-based models, light interception is classically represented through the Beer–Lambert–Bouguer law, hereafter named as Beer-Lambert (BL), assuming a simple exponential relationship to describe light attenuation within a homogeneous canopy [Monsi and Saeki, 1953, cited in English translation in Hirose (2005)]. It relies on a simple one-dimensional turbid medium model, which raises several limitations regarding its consistency with experimental data (Ponce de León and Bailey, 2019), its stability in relation to other environmental variables (Valladares et al., 2012), and the vertical variation of leaf photosynthetic parameters in the canopy (Sarlikioti et al., 2011), as well as its theoretical foundations (Kostinski, 2002). In particular, this equation assumes that foliage is randomly dispersed, a hypothesis that, depending on the species architecture, can lead to over-estimation of light interception if the foliage is clumped or, on the contrary, under-estimation if the plant plasticity allows optimizing foliage distribution for an enhanced light interception [e.g., for beech tree (Schröter et al., 2012)]. In order to overcome these problems, other approaches have been proposed recently, notably that of (Shabanov and Gastellu-Etchegorry, 2018) and Casasanta and Garra (2018). In Shabanov and Gastellu-Etchegorry, 2018, the authors derive a stochastic formulation of the BL law, which accounts for heterogeneous canopies. Their virtual experiments reveal that the traditional law is not universally applicable across different canopy structures. In Casasanta and Garra (2018), the authors introduce two stochastic approaches to the problem. The first one is based on a fractional Poisson process, resulting in a *fractional* BL law based on the Mittag-Leffler function, also discussed later in the present study (see Eq. 16). The second is based on weighted Poisson distributions, resulting in a Mittag-Leffler weighted BL law. In line with their work, we also propose some possible generalizations of the BL law. In particular, by modeling appropriately the probability of the event of interception, we derive a class of models for water consumption prediction.

Therefore, the main objective of our study was to explore to which extent some mechanistic principles borrowed from physiologically based models could be incorporated into more statistical approaches for predicting the water consumption of plants. To this end, our approach was *(i)* to analyze the identifiability of some compartmental simplifications of the GreenLab model for tomato plants in the case where the data set consists only of environmental variables, *(ii)* to derive from the GreenLab principles a new family of models focusing on water consumption as the main state variable and differing by their assumptions regarding the dynamics of the LIR, and *(iii)* to perform a very preliminary comparison of these models using an experimental dataset of water consumption by tomato plants. Although mostly theoretical at this stage, this work has some conceptual interest in presenting an original stochastic approach to derive a new class of simple models and providing a procedural guideline to further confrontations to experimental results.

## 2 Materials and methods

### 2.1 Data collection

Between May 10 and July 2, 2021, an extensive study was conducted in a hydroponic greenhouse near Therma village, within the Nigrita-Serres region (40.91, 23.55), Greece, to examine the tomato plant’s (cv. ecstasis) water consumption patterns. A drip irrigation system was used to ensure precise irrigation for each individual plant. Rockwool was used as a substrate growing medium, a product of basalt mainly composed of Oxide of Calcium (*CaO*) with small percentages of Iron (*Fe*) and Aluminum (*Al*), in keeping with common practices in the region. Plants’ density is reported as 5 stems per *m* ^2^ (one stem per plant). Indoor measurements were performed using an Efento Logger (Efento, 2020). Additionally, meteorological data were collected using a Davis Vantage Pro 2 (Plus) weather station close to the greenhouse. A comprehensive overview of the measured quantities, including Solar Radiation, Temperature, Humidity, and Air pressure, averaged on a daily level, is presented in **Table 1**.

**Table 1:** Summary of all measured variables (units in parenthesis) in column 1.

Variables	Mean	St. Dev.	Min	Max
Avg Solar Radiation (*W/m* ^2^)	286.944	45.844	114.590	340.310
Max Solar Radiation (*W/m* ^2^)	1,021.830	131.702	571	1,329
Avg Air pressure (hPa)	1,014.045	3.601	1,007.120	1,022.720
Avg Temperature (*°C*)	22.043	2.642	17.060	28.630
Max Temperature (*°C*)	29.306	3.086	23.100	37.800
Min Temperature (*°C*)	15.707	3.403	9.100	22.000
Avg Humidity (%)	0.837	0.109	0.618	0.976
Max Humidity (%)	0.984	0.031	0.840	1.000
Min Humidity (%)	0.591	0.161	0.230	0.860
Water Consumption (L)	1.102	0.519	0.090	2.250

The most basic descriptive statistics (mean, sd, min, max) from a sample of N = 54 measurements are given in columns 2-5.

Initially, the plants were grown in a location separate from the greenhouse before being relocated to the designated study area. The period spanning from seed planting to the onset of observations was 38 days. Upon arrival, the plants were observed to be in the initial stage of their first inflorescence, i.e., for the majority of plants, the first truss has just been formed. The measurements were thus performed from day 39 to day 92 of the plant growth, from 10/5/2021 to 2/7/2021, for a total of 54 days. Tomato plants are usually grouped into stations, each combining a substrate (slab) with *n* plants and a pot for water collection. The per average plant volume of water consumed by a station is referred to as *Water Consumption* (*W_c_*), given by


(1) $$W_{c}\left(t\right)=\frac{V_{Irr}\left(t\right)-R_{of\;f}\left(t\right)}{n},$$

where *V_Irr_* (*t*) is the volume (*L*) of applied water at time *t*, and *R_of f_* (*t*) the volume (*L*) of the corresponding collected excess water which accumulates from the application time until the next morning during 12 hours. In our study, *n* = 3, and the Water Consumption (*W_c_*) corresponds to the dependent variable we try to estimate and predict.

### 2.2 Brief presentation of the GreenLab model for tomato

The GreenLab model has been extensively explored in the literature, see, e.g., Dong et al. (2008) and Zhang et al. (2009) for its application on tomato. Hereafter, we briefly recall its main principles, and the interested reader is referred to **Appendix 1.1** for a more comprehensive description of the model and to **Table 2** for the specific parameter values that we used in this work.

**Table 2:** Parameters of the GreenLab model for tomato and their values in our study.

Parameter	Comments	value
*p_b_*	Blade relative sink strength	1
*p_p_*	Petiole relative sink strength	1.09
*p_e_*	Internode relative sink strength	0.93
*p_f_*	Individual fruit relative sink strength	61.3
*B_b_*	Blade sink variation parameter	0.43
*B_p_*	Petiole sink variation parameter	0.45
*B_e_*	Internode sink variation parameter	0.38
*B_f_*	Fruit sink variation parameter	0.36
*S_p_*	Projection surface (cm^2^)	5047
*k*	Beer Lambert coefficient	0.8
RUE	Radiation Use Efficiency	0.05
*T_b_*	maximum expansion time of blade (CDs)	10
*T_e_*	maximum expansion time of internode (CDs)	8
*T_p_*	maximum expansion time of petiole (CDs)	10
*T_f_*	maximum expansion time of fruit (CDs)	15
phyllocron	elapsed time between two leaves emergences (days)	2

Plant development is assumed to be deterministically driven by the rules of a parameterized automaton which determines the sequential appearance of phytomers (plant species-specific combinations of organs) and their respective positions. The thermal time elapsing between the appearance of two successive phytomers, assumed to be constant, serves as the discrete (simulation) time step and is referred to as a Cycle of Development (CD). The organogenesis depends solely on thermal time, triggered above a base temperature of 12*°C* (Shamshiri et al., 2018).

The structure of a tomato plant can be delineated by four types of primary organs (excluding roots): *blade* (b), *petiole* (p), *internode* (e), and *fruit* (f). Following the simplifications proposed by Dong et al. (2008) and Zhang et al. (2009), we considered flowers and fruits as the same organ (i.e. the dynamics of biomass allocation do not distinguish the transition from flower to fruit). Typically, in the cultivation of single-stem, pruned, tomato varieties within greenhouses, seven to eleven phytomers devoid of flowers develop prior to the emergence of the first inflorescence. In the present study, it is assumed that, following the first eight phytomers without flowers, a truss appears at every third phytomer, producing three flowers that bud. This specific assumption is consistent with empirical evidence from the present study and translates to an average of three fruits per truss.

The integration of photosynthetic production is calculated using the Beer-Lambert (BL) law (Monteith, 1977; Hirose, 2005), which is analogous to the approach employed in most process-based models:


(2) $$Q\left(t\right)=E\left(t\right)\cdot \text{RUE}\cdot S_{p}\cdot \left(1-\text{exp }\left\{-k\;S_{L}\left(t\right)/S_{p}\right\}\right),$$

where during CD(*t*), *Q*(*t*) corresponds to the newly synthesized (dried) biomass, *E*(*t*) to the Solar Radiation, and RUE is the Radiation Use Efficiency (the vegetation efficiency of converting radiative energy into biochemical energy through photosynthesis). Moreover, *S_p_* represents the projected surface potentially occupied by a single plant, while *S_L_* (*t*) stands for the plant’s photosynthetically active leaf area, calculated as the sum of the total photosynthetically active biomass of the blades multiplied by the specific leaf area (*SLA*: coefficient converting a unit of produced biomass to leaf surface). The variable *k* corresponds to the extinction coefficient in the Beer-Lambert law, and it is set to 0.8 for the tomato crop, as in Zhang et al. (2009). For *t* = 0, the initial biomass of the seed is denoted by *Q*_0_.

At each CD, the available biomass is shared between all growing organs of the plants, regardless of their spatial position and proportionally to their current sink strength, according to the so-called common pool assumption that was investigated for tomato by Heuvelink, 1995. The growth Δ*q_o_* (*u, t*) of an organ of type *o* and chronological age *u* (days or CDs), while the plant is in cycle *t* ≥ *u*, can then be expressed as:


(3) $$\text{Δ}q_{o}\left(u,t\right)=p_{o}\cdot f_{o}\left(\frac{u}{T_{o}}\right)\cdot \frac{Q\left(t-1\right)}{D\left(t\right)},$$

where *p_o_* is the relative sink strength of the organ of type *o*, *f_o_* (·) its sink variation function related to the organ’s biomass demand profile during its expansion and *D*(*t*) the total demand in cycle *t* (see Eq. 23). As in Yan et al., 2004, *f_o_* (·) corresponds to a discretized beta law function with shape parameters *a_o_* and *b_o_* (see Eq. 26). For identifiability reasons, discussed in Dong et al., 2008, the constraint *a_o_* + *b_o_* = 5 is imposed for tomato plants.

### 2.3 Link with water consumption

Howell and Musick (1985) demonstrated that transpiration and biomass production are proportional in their set of environmental conditions that encompasses our experimental conditions (**Table 1**) (Howell et al., 1984). In our greenhouse setting, evaporation is assumed to be negligible, so transpiration could, in turn, be considered proportional to water consumption (Food and Agriculture Organization of the United Nations, 1998), thus rendering the latter linearly related to dry matter production. Disregarding evaporation is not a particularly far-fetched premise within the framework of hydroponic greenhouses. These greenhouses are designed to reduce evaporation to a minimum, utilizing substrates wrapped in white sacks that offer a minimal surface area for water to evaporate from (Resh, 2022).

Adding normally distributed homoskedastic errors, we obtain the following initial model:


(4) $$W_{c}\left(t\right)=\mu_{0}\cdot Q\left(t\right)+\varepsilon_{t},\text{ where }\varepsilon_{t}∼N\left(0,\sigma^{2}\right),$$

where *µ*_0_ is a positive proportionality constant and *σ* ^2^ is a variance parameter representing the experimental variability of the measurement process.

As *W_c_* measurements were conducted daily, but the GreenLab model runs on Cycles of development (CD), we need to map CDs on days. Elapsed days between two successive leaf developments (phyllochron) can vary from 1.5 (summer) to 3 (autumn) days according to the genotype, and the climatic conditions (Pivetta et al. (2007); Schmidt et al. (2017)). We assume that the phyllochron is stable and equal to 2 days, as we measured a mean value of 10*°Cd* with a base temperature of 12*°C*. To aggregate the two separate measurements into one CD, a weighted average is utilized with a weight proportional to the fraction of the Solar Radiation of each day.

### 2.4 Identifiability issues and compartmental simplification of the GreenLab model

In our realistic setting, where no plant data are available, estimating the parameters of the complete Greenlab model is unfeasible since identifiability problems typically arise: it means that different sets of parameter values generate the same simulated dynamics, for a specified set of output variables. Thus, in our case, plants with different characteristics could have the same dynamics of water consumption.

Adopting a general dimensionality reduction strategy for non-identifiability issues—outlined in Hastie et al. (2009)—we analyzed a simplified version of the model. We trade precision in representing the biological model for enhanced identifiability within the parameter space. In this version, we combined all the biomass of petioles (*p*), internodes (*e*), and fruits (*f*) into a single representative referred to as *body*.

Parameters requiring estimation thus comprise:


(5) $$\theta =\left(a_{b},\;b_{b},\;p_{body},\;a_{body},\;b_{body},\;S_{p},\text{ RUE},\;SLA,\;\mu_{0},\;\sigma ,\;Q_{0}\right)$$

We will refer to this specific parametrization as *comp1*.

To explore the identifiability of parameters we performed simulation experiments under realistic scenarios. It consists of generating virtual observations from a realistic set of parameter values and investigating which parameters can be accurately estimated: non-identifiability is revealed when the estimated values are significantly different from the ones used for simulating the observations. In that case, the corresponding parameters should be set as a constant and removed from the list of parameters to estimate, in order to reduce the dimension of the parameter vector until reaching an identifiable subset.

For the sake of simplicity, we present in Section 3.1 the results from two characteristic cases only, which correspond to the *comp1* model. In the first one, we fix *SLA*, the specific leaf area, and *Q*_0_, the initial biomass of the seed, quantities that can typically be measured. Parameters *S_p_* and RUE are also fixed, since we incorporated the *µ*_0_ parameter in the model 4, a simplification justified by the compensation effect between those parameters. In the second case, additionally to the previously mentioned parameters, we fix *P_body_*, the sink strength of the “body” compartment. By initializing 5000 randomly selected starting points, we recorded the solutions that maximize the likelihood function of the model, with a tolerance of < 10^−3^ to account for numerical approximations. The maximization of the function was performed via a BFGS (Broyden–Fletcher–Goldfarb–Shanno) quasi-Newton algorithm for Bound Constrained Optimization (Byrd et al., 1995).

### 2.5 Two model versions for water consumption time series based on the recurrence equation of GreenLab

As shown in Letort et al. (2009), the GreenLab model can be synthesized into a single recurrence equation that, for the sake of simplicity, we chose here to formulate as:


$$Q\left(t\right)=E\left(t\right)\cdot \text{RUE}\cdot S_{p}\left(1-\text{exp }\left\{-\frac{k\cdot SLA}{S_{p}}\;\sum_{n=0}^{t-1}r\left(n\right)Q\left(n\right)\right\}\right),$$

where *r*(*n*) represents the proportion of green to the totally produced biomass *Q*(*n*), a quantity that can be calculated as a function of the model parameters. Assuming proportionality (with constant *µ*_0_) between biomass production and water consumption and no leaf senescence, we obtain a general model form for water consumption:


(6) $$W_{c}\left(t\right)=\theta_{1}\cdot E\left(t\right)\cdot \left(1-\text{exp }\left\{-\theta_{2}\;\sum_{n=0}^{t-1}r\left(n\right)W_{c}\left(n\right)\right\}\right),$$

where *θ*_1_ = RUE·*S_p_* ·*µ*_0_ and $\theta_{2}=\frac{k\cdot SLA}{S_{p}\cdot \mu_{0}}$ are estimated, while the other parameters which appear implicitly in the coefficients *r*(*n*) are fixed at the values found in Dong et al. (2008) (see **Table 2**). This model will be referred to as *GreenLab exp*.

To account for the obviously existing differences between the tomato plants in Dong et al. (2008) and those available in this study, we propose a modified parametric version of the coefficients as follows:


$$r\left(t\right)=\frac{t^{a}}{I\left(a\right)},\text{ where }I\left(a\right)=\int_{0}^{t_{max}}t^{a}\;dt=\frac{t_{max}^{a+1}}{a+1}$$

corresponds to a normalization constant with respect to *a*, a parameter to estimate, and to the maximum time of observation *t_max_*. This model will be referred to as *exp + rate*.

### 2.6 Stochastic models of light interception to predict water consumption

Building upon the prior discussion, we now focus on a novel aspect that broadens the model formulation. Here, we aim to represent biomass production at time *t*, as the cumulative byproduct of a composite stochastic experiment, which consists of many independent individual experiments, each one deciding

whether elementary radiative inputs will be absorbed by the plant or not. We thus derive a family of models, which will be referred to as “Stochastic Segmentation of input Energy” models (SSiE).

#### 2.6.1 Formulation of the water consumption series from a stochastic model of light interception

In this section, we discuss the intuition behind a probabilistic interpretation of biomass production, and we formalize this intuition with tools from theoretical probability. At each time *t*, a total radiative input *E*(*t*) is channeled into the system per *m* ^2^. We assume that this input is equally quantized into very small elementary quantities ${\left\{E_{i}(t)\right\}}_{i=1}^{n}$ in such a way that either they are completely absorbed by the plant and converted into biomass by the enlightened parts of the plant or they exit the system without affecting it. In this case, *E_i_* (*t*) = *E*(*t*)*/n* where *n* represents the number of “elementary” units. If no other specific details are known, one could assume that the individual events of absorption, say *A_i_* (*t*), are independent with identical probability of occurrence *p*(*t*). With this interpretation and if 1*_A_i_(t_*_)_ stands for the indicator function of the corresponding event, each elementary radiative input *E_i_* (*t*) is associated with a random variable


(7) $$Q_{i}\left(t\right)=\text{RUE}\cdot S_{p}\cdot E_{i}\left(t\right)\cdot 1_{A_{i}\left(t\right)},$$

which records its produced biomass, either 0 if the event *A_i_* (*t*) is not realized or RUE · *S_p_* · *E_i_* (*t*) if the event is realized, and thus it is totally transformed. The total biomass produced by the plant at time *t* can thus be expressed as follows:


(8) $$Q^{\left(n\right)}\left(t\right)=\sum_{i=1}^{n}Q_{i}\left(t\right)=\text{RUE}\cdot S_{p}\cdot E\left(t\right)\cdot \frac{\sum_{i=1}^{n}1_{A_{i}\left(t\right)}}{n}.$$

Clearly, the last factor of the above expression corresponds to the sample mean of independent and identically distributed random variables and in particular Bernoulli random variables with common probability *p*(*t*). Intuitively, one should expect by the strong law of large numbers that the sample mean value should be very near to their common probability of absorption, that is, *p*(*t*). These arguments give an intuitive interpretation of the fact that the following approximations should be plausible:


(9) $$Q\left(t\right)\approx Q^{\left(n\right)}\left(t\right)\approx \text{RUE}\cdot S_{p}\cdot E\left(t\right)\cdot p\left(t\right).$$

However, despite the seemingly sound arguments underlying these approximations, a theoretical justification of their validity is more complex. An obvious theoretical caveat regarding the validity of these approximations is that we cannot conceptualize a countably infinite sequence of events of common probability that play the role of the elementary events of biomass absorption, or equivalently the total radiative input cannot be partitioned into a countably infinite number of positive parts potentially transformed into biomass. One possibility for justifying the above approximations would be to resort to an uncountable number of stochastic experiments. This approach involves more mathematical intricacies. For this reason, and since a rigorous justification of this part is not necessary for the rest of the paper, the interested reader is referred to **Appendix 1.2** for more details.

The next step is to appropriately model the probability of absorption *p*(*t*), which can classically be done through a parametric family of continuous distribution functions. For each time $t,\text{ let}{\left\{Z_{u}(t)\right\}}_{u=0}^{E(t)}$ represent the Bernoulli experiments of absorption of the radiative input for all possible *u* ranging from 0 to *E*(*t*). If we denote by *LIS*(*t*) the Light Interception Surface at time *t*, then, assuming that the maximum available soil surface is *S_p_*, one could construct a new family of random variables ${\left\{U_{u}(t)\right\}}_{u=0}^{E(t)}$ uniformly distributed on [0*,S_p_*] which concretize the above experiments. In particular, the interval [0*,S_p_*] is partitioned into two subintervals [0*,LIS*(*t*)] and (*LIS*(*t*)*,S_p_*]. Then, the absorption events can be written as


(10) $$A_{u}\left(t\right):=\left\{Z_{u}\left(t\right)=1\right\}=\left\{U_{u}\left(t\right)\leq LIS\left(t\right)\right\},0\leq u\leq E\left(t\right).$$

In probability theory, such a family exists; loosely speaking, this reinterpretation of the absorption events corresponds to a collection of idealized experiments where an elementary radiative input enters into the system if it intersects with the green part of the plant. Now, notice that *p*(*t*) corresponds exactly to the probability of the event given by (10), which is related to the Light Interception Surface *LIS*(*t*) at time *t*. However, *LIS*(*t*) is not directly observable, but only indirectly via the cumulated water consumption prior to time *t*, denoted by *SW_c_* (*t* ^−^) (itself proportional to the cumulated produced biomass). A novelty of this study consists in making a link between *LIS*(*t*) and *SW_c_* (*t* ^−^) through an increasing (non-decreasing) function *g*: ℝ_+_ →ℝ_+_, that is, *LIS*(*t*) = *g*(*SW_c_* (*t* ^−^)). By the above argument, Eq. (10) and the fact that *U_u_* (*t*) ∼ Unif(0*,S_p_*) we get that all the following equalities hold:


$$p\left(t\right)=\mathbb{P}\left(U_{u}\left(t\right)\leq LIS\left(t\right)\right)=\mathbb{P}\left(U_{u}\left(t\right)\leq g\left(SW_{c}\left(t^{-}\right)\right)\right)=\frac{g\left(SW_{c}\left(t^{-}\right)\right)}{S_{p}}=\frac{LIS\left(t\right)}{S_{p}}≕\;LIR\left(t\right),$$

where the last term stands for the Light Interception Ratio. Now, also notice that if *U* ∼ Unif(0*,S_p_*) is a copy from the family ${\left\{U_{u}(t)\right\}}_{u=0}^{E(t)}\text{and }g$ is invertible, then the third term above can be rewritten as


(11) $$LIR\left(t\right)=\mathbb{P}\left(g^{-1}\left(U\right)\leq SW_{c}\left(t^{-}\right)\right)=\mathbb{P}\left(X\leq SW_{c}\left(t^{-}\right)\right)=F_{X}\left(SW_{c}\left(t^{-}\right)\right),$$

where we set *X* = *g* ^−1^(*U*). In fact, since *g* is assumed to be an increasing function, its inverse exists at least in a generalized form (generalized inverse) and the above equations still hold. The problem is then to define the relationship between *LIR*(*t*) (or *LIS*(*t*)) and *SW_c_* (*t* ^−^) without having any information on the plant itself and in the next section we discuss several such possibilities.

#### 2.6.2 Different options for the distribution of X

The determination of a mechanistic functional relationship between *LIR*(*t*) and *SW_c_* (*t* ^−^) is unrealistic. Biologically speaking, the underlying processes are complex and involve, among others, the patterns of biomass allocation to blades and their arrangement in space. An approach to this objective is, however, feasible and a selected number of possible distribution families could be used to compete for their fitting quality and their predictive ability. By introducing additive errors as in Section 2.3, we can derive a model directly applicable to the Water Consumption variable


(12) $$W_{c}\left(t\right)∼N\left(\;\theta_{1}\cdot E\left(t\right)\cdot F_{X}\left(SW_{c}\left(t^{-}\right)\right),\;\sigma^{2}\;\right),$$

thereby eliminating the requirement for biomass as intermediary variable. Each model is determined by specifying *F_X_* in one of the following parametric family of distributions.

**Exponential distribution**

The exponential distribution is one of the most fundamental suppositions that one can make when faced with an undetermined distribution, since it corresponds to the maximum entropy solution for a given expected value on the positive line (Jaynes, 1957). Besides, in our setting, it leads to a Beer-Lambert-like model. By (11) and the assumption of an exponential model, we get:


(13) $$LIR\left(t;k\right)=1-\text{exp }(-k\cdot SW_{c}\left(t^{-}\right)),t\geq 0.$$

**Gamma distribution**

The gamma distribution is a generalization of the exponential distribution. This provides a logical progression from our initial assumption of an exponential distribution. By (11) and the assumption of a gamma model, we get:


(14) $$LIR\left(t;k,a_{\gamma }\right)=\int_{0}^{SW_{c}\left(t^{-}\right)}\frac{k^{a_{\gamma }}}{\text{Γ}\left(a_{\gamma }\right)}\;s^{a_{\gamma }-1}\;e^{-k\cdot s}\;ds,\;t\geq 0.$$

**Mittag-Leffler distribution**

Mittag-Leffler introduced the function bearing his name in 1903 (Bateman, 1953). Different properties of the distribution generated by the Mittag-Leffler function were explored in Pillai (1990). The concept of generalizing the Beer-Lambert law with the use of the Mittag-Leffler function was proposed by Casasanta and Garra (2018). Following their work, we incorporate this generalization into our analysis, leading to the following LIR term:


(15) $$LIR\left(t;k,a_{ML}\right)=1-E_{a_{ML}}(-\left(k\cdot SW_{c}\left(t^{-}\right){)}^{a_{ML}}\right),\;t\geq 0,$$

where *E_aML_* is the Mittag-Leffler function:


(16) $$E_{a_{ML}}\left(x\right)=\sum_{j=0}^{\infty }\frac{x^{j}}{\text{Γ}\left(j\cdot a_{ML}+1\right)},\;x\in \mathbb{R},$$

with *a_ML_* ∈ (0,1] the *tail* parameter and *k >* 0 the *rate* parameter. For *a_ML_* = 1 the above formulation reduces to the exponential distribution with rate parameter *k*.

**Log-normal distribution**

The log-normal distribution is commonly employed to model growth rates. Our reasoning for incorporating this distribution in our analysis stems from the presumption that the elementary events ${\left(A_{i}\right)}_{i=1}^{n}$ are influenced by the incremental growth of smaller plant elements. This growth is contingent on their size. For the density function, we proceed by adopting the ensuing parametrization:


(17) $$LIR(t;\mu_{\log},\sigma_{\log})=\int_{0}^{SW_{c}(t^{-})}\frac{1}{s\cdot \sigma_{\log}\cdot \sqrt{2\pi }}\exp\left(-\frac{(\log(s)-\mu_{\log}{)}^{2}}{2\sigma_{\log}^{2}}\right)\;ds,\;t>0.$$

**Pareto distribution**

The last distribution we explore is Pareto. Following Van der Zande et al., 2010 (mainly the results depicted in Figures 2, 3), we observe that the percentage of the biomass responsible for most of the energy interception follows a similar law to the Pareto 80*/*20 rule (Juran and De Feo, 2010). The formulation of the distribution function that we adopt is as follows:


(18) $$LIR\left(t;\theta ,\eta \right)=1-{\left(\frac{\eta }{SW_{c}\left(t^{-}\right)}\right)}^{\theta },\;SW_{c}\left(t^{-}\right)>\eta .$$

### 2.7 Model comparison and prediction performance criteria

To have a challenging baseline model to compete with, we first estimated a linear first-order autoregressive model with one exogenous variable, namely the average solar radiation received at day *t*, *E*(*t*):


(19) $$W_{c}\left(t\right)=b_{0}+b_{1}W_{c}\left(t-1\right)+b_{2}E\left(t\right)+\varepsilon_{t},$$

where the *b_i_* coefficients are estimated via the ordinary least squares method.

In terms of forecasting, a sequential methodology is employed. From the original dataset, we initially extract the first 55% days (days 39 to 68) for training and predict the next day’s water consumption (day 69). Subsequently, we increase the size of the training set by one additional day at each step, continuing to predict the following day until we reach the end of the time series. The parameters are re-estimated at each step of the procedure, using a total of 1000 distinct starting points in our calculations, subsequently selecting the point with the highest likelihood value as the model’s parameter for prediction. However, in the case of the *ml f* model, the number of initial points was reduced to 20, to reduce the computational burden. After the parameter estimation process, a model selection procedure was performed with the two most classical model selection criteria, namely the corrected Akaike Information Criterion (AICc) (Hurvich and Tsai, 1989) and the Bayesian Information Criterion (BIC).

The setting described above reflects real-world conditions as it emulates the practical scenario where we have a bunch of observations, and our objective is to forecast Water Consumption for the upcoming day. Two different settings were considered for the inputted Solar Radiation (E): (i) assumed to be perfectly known (fixed covariate setting), or (ii) with an additive white noise factor associated with predicting solar radiation, where the standard deviation was set empirically at 20, a value that corresponds to a bound on values typically obtained with current prediction models (Tao et al., 2019).

The predictive performance of the models was compared with the Root Mean Square Prediction Error (RMSPE):


(20) $$\text{RMSPE}\left(\hat{y}\right)=\sqrt{\frac{1}{m}\sum_{i=1}^{m}{\left(y_{i}-\hat{y_{i}}\right)}^{2}},$$

where *y* = (*y_i_*) is the vector of observed values and *ŷ* = (*ŷ_i_*) the predicted ones. For the testing set, according to the previously described protocol we used *m* = 24 observations. The computer programs were developed in R (version 4.3.1.) and the packages *MittagLeffleR* (Gill and Straka, 2018) and *tidyverse* (Wickham et al., 2019) were used for computations with the Mittag-Leffler distribution and other data manipulations and visualization respectively.

## 3 Results

### 3.1 Identifiability analysis of the GreenLab model with compartmental simplification

**Figure 1:**
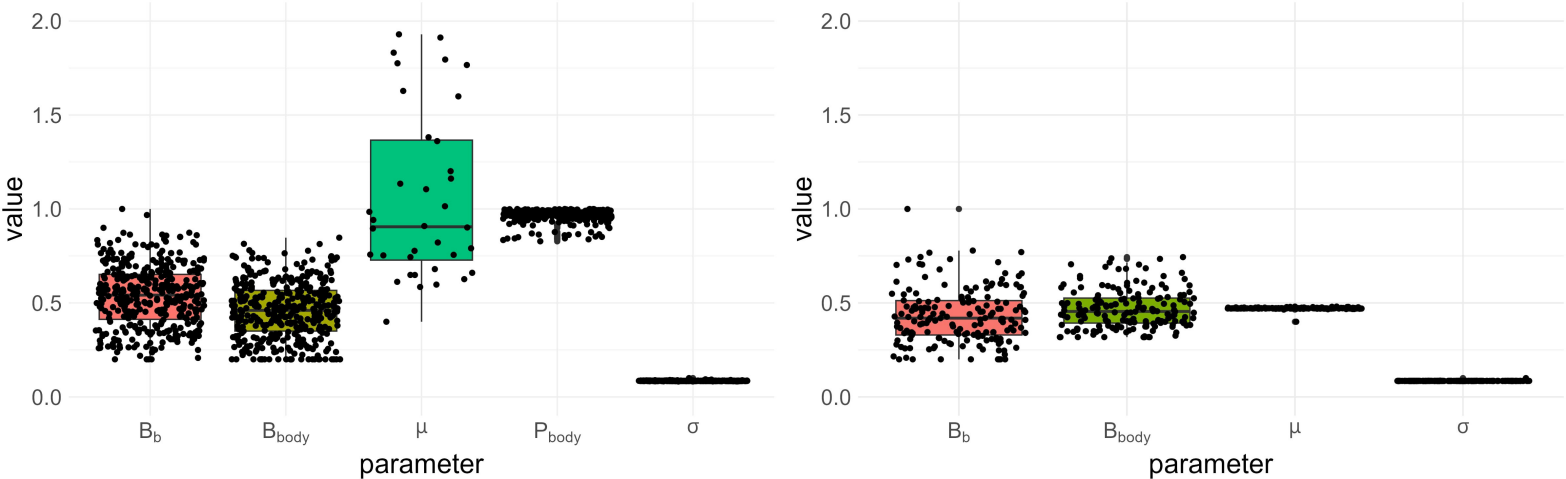
Boxplots of estimated values with similar likelihood for the two cases presented in Section 3.1. Each dot represents an estimated value. The sink strength of the body compartment (*P_body_*) is normalized by its maximum for scaling reasons. (Left) Fixed parameters: *SLA*, *S_p_*, RUE, *Q*_0_. (Right) Fixed parameters: *SLA*, *S_p_*, RUE, *Q*_0_, *P_body_*.

When considering only water consumption data, a certain number of the GreenLab parameters are not identifiable. This is true even when the simplified and parsimonious *comp1* model is used which has fewer parameters than the complete one (Section 2.4). The boxplots in **Figure 1** allow comparing the case where *P_body_* is estimated (in addition to *B_b_*, *B_body_*, *µ*_0_, and *σ*) with the case where it is set at its reference value. Each point represents an estimated parameter value, and specific combinations of these points correspond to the estimated solutions of the maximization problem. Note that, for scaling purposes, *P_body_* has been normalized by its maximum value. The plots reveal that many distinct solutions yield similar likelihood values. As can be seen by comparing the ranges of the estimated parameters (see **Figure 1**, left and right), this identifiability issue diminishes as we set more parameters, but never disappear. Even with only four estimated parameters, we remark compensation effects between *B_b_* and *B_body_*, since the resulting estimates still vary significantly. However, the parameters *µ*_0_ and *σ* are identifiable, at least locally, around the chosen reference values, a noteworthy result which enables the elaboration of the stochastic framework discussed in Section 2.6.

### 3.2 Estimation of the linear and SSiE models’ parameters

The regression results for the linear autoregressive model 19 with Average Solar Radiation as an exogenous variable show that all parameters appear to be statistically significant at a 0.01 significance level (**Table 3**). The coefficient of determination *R* ^2^ and the adjusted one have similar values, of approximately 0.88.

**Table 3:** Summary of the base linear regression model of Water Consumption vs the predictors given in the first column (units in parenthesis).

	*Dependent variable:*
	*W_c_* (*t*) (*L*)
Avg Solar Radiation (*W/m* ^2^)	0.002 (0.001) ^∗∗∗^
*W_c_* (*t* − 1) (*L*)	0.893 (0.051) ^∗∗∗^
Constant	−0.480 (0.156) ^∗∗∗^
Observations	53
R^2^	0.884
Adjusted R^2^	0.879
Residual Std. Error	0.178 (df = 50)
F Statistic	189.652^***^ (df = 2; 50)
*Note:*	^*^p<0.1; ^**^p<0.05; ^***^p<0.01

The estimated coefficients (sd in parenthesis) are given in the second column. Asterisks denote the statistical significance according to Student’s t-test.

The estimated parameter values of each SSiE model (**Table 4**) and their relative performances (**Table 5**) according to the comparison criteria defined in 2.7 highlight a slight superiority of the *lognormal* and *pareto* models in terms of both the Bayesian Information Criterion (BIC) and the corrected Akaike Information Criterion (AICc). A straightforward application of Eq. 6 by estimating the green biomass by an already fitted model (Dong et al., 2008) does not appear to be highly promising, as it still results in higher values in these criteria. Similar behavior is present in the Beer-Lambert-like approach of the exponential distribution.

**Table 4:** Estimated parameters for the models described in (6) and (12) (see Section 2.6.2).

Version	*θ*_1_	*σ*	*k* or *θ*_2_	*α*	*μ_log_*	*σ_log_*	*θ*	*η*
lognorm	0.011	0.165	–	–	3.958	9.273	–	–
pareto	370.112	0.165	–	–	–	–	3.02·10^-6^	0.403
mlf	0.01	0.166	0.017	0.501	–	–	–	–
gamma	0.007	0.169	0.01	0.386	–	–	–	–
exp +rate	0.007	0.172	2.037	-0.834	–	–	–	–
GreenLab exp	0.005	0.208	0.559	–	–	–	–	–
exp	0.005	0.211	0.133	–	–	–	–	–

The pair (k, θ_2_) is aligned in the same column. "-" denotes that the parameter of the model represented by the row does not include the parameter, represented by the column. In short the model does not use this parameter.

**Table 5:** Comparison of different distribution choices regarding the formulations in (6) and in (12).

	Version	log lik val	RMSE	# param	BIC	AICc
1	lognorm	20.45	0.16	4	**-25.02**	-31.62
2	pareto	20.39	0.16	4	**-24.9**	-31.5
3	mlf	19.85	0.17	4	-23.82	-30.42
4	gamma	19.12	0.17	4	-22.36	-28.96
5	exp + rate	18.15	0.17	4	-20.42	-27.02
6	LR	17.7	0.17	3	-19.51	-26.57
7	GreenLab exp	8.14	0.21	3	-4.37	-9.45
8	exp	7.25	0.21	3	-2.59	-7.67

The columns refer successively to the method’s name, the estimated log-likelihood value (log lik val), the RMSE, the total number of estimated parameters, the BIC and the AICc criteria. Bold lines indicate the "best" values according to the criterion under consideration.

A notable result is the estimation of *a_ML_* ≃ 0.5 (see **Table 4**). For *a_ML_* = 0.5 the Mittag-Leffler function (16) reduces to (Haubold et al., 2011):


$$E_{1/2}\left(x\right)=e^{x^{2}}\;\left(1-\frac{2}{\sqrt{\pi }}\int_{0}^{x}e^{-s^{2}}\;ds\right),$$

where $\frac{2}{\sqrt{\pi }}\int_{0}^{x}e^{-s^{2}}\;ds$ , also known as the Gauss error function, is a quantity which expresses the probability of a typical Gaussian distribution to be found in the interval [−*x,x*] for *x* ≥ 0. In our case this translates to:


$$LIR\left(t\right)≃1-\text{exp }\left(-k\cdot SW_{c}\left(t^{-}\right)\right)\cdot \mathbb{P}\left(\left|Z\right|>\sqrt{k\cdot SW_{c}\left(t^{-}\right)}\right),\;t\geq 0,$$

where *Z* ∼ *N*(0,1). Another noteworthy finding is related to the pareto model and specifically the parameter *η* which corresponds to the initial cumulative water consumption *SW_c_* (*t*) up to the first observation time. This parameter was estimated at 0.403 (see **Table 4**) and corresponds approximately to 400*ml* over a span of 38 days.

**Figure 2:**
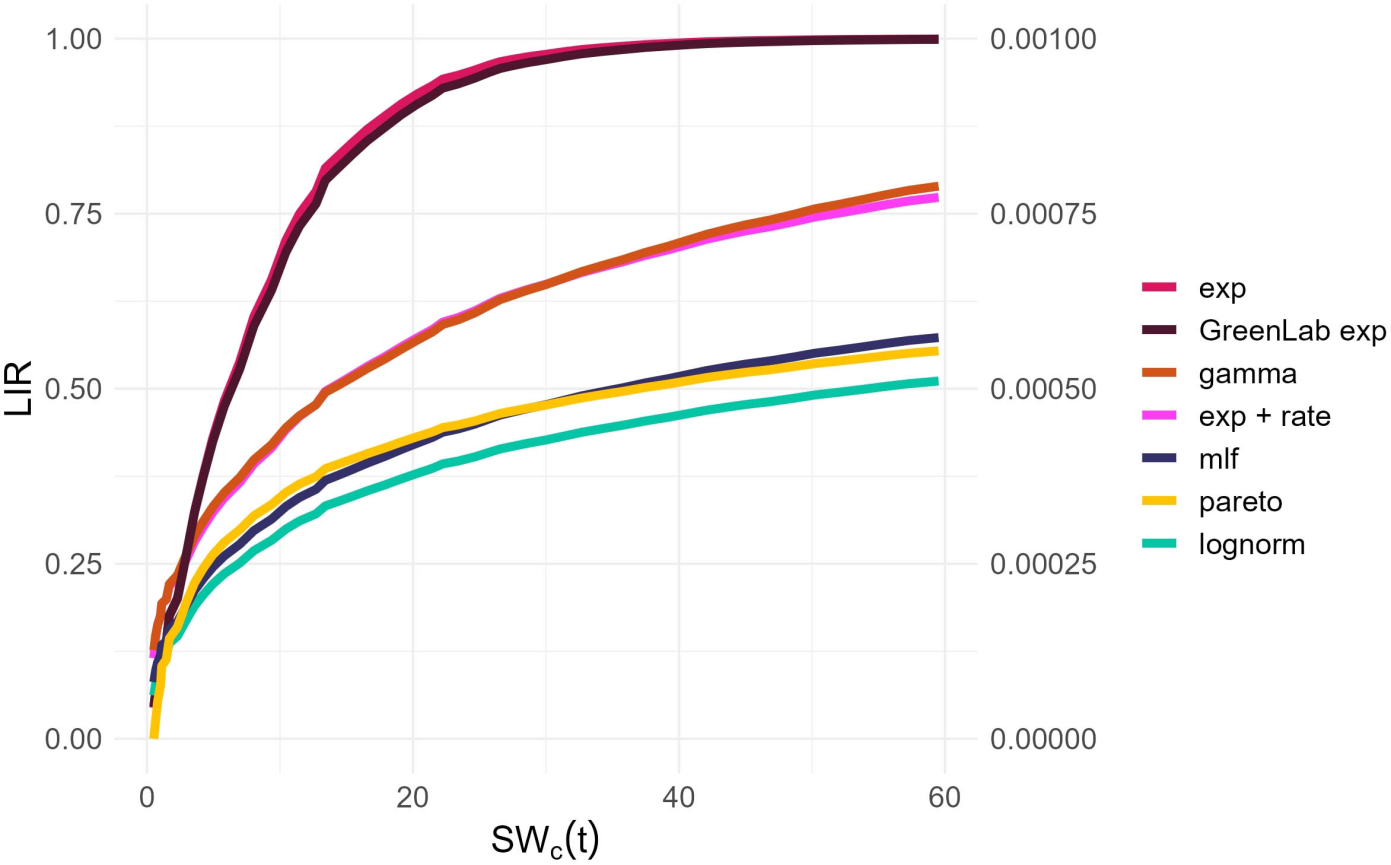
Estimated LIR from the competing models (2.6.2) as a function of the accumulated water usage. The right axis was included for the values of the Pareto distribution. The *Lognorm* and *GreenLab exp* overlap, as well as the *Gamma* and the *LR*.

**Figure 3:**
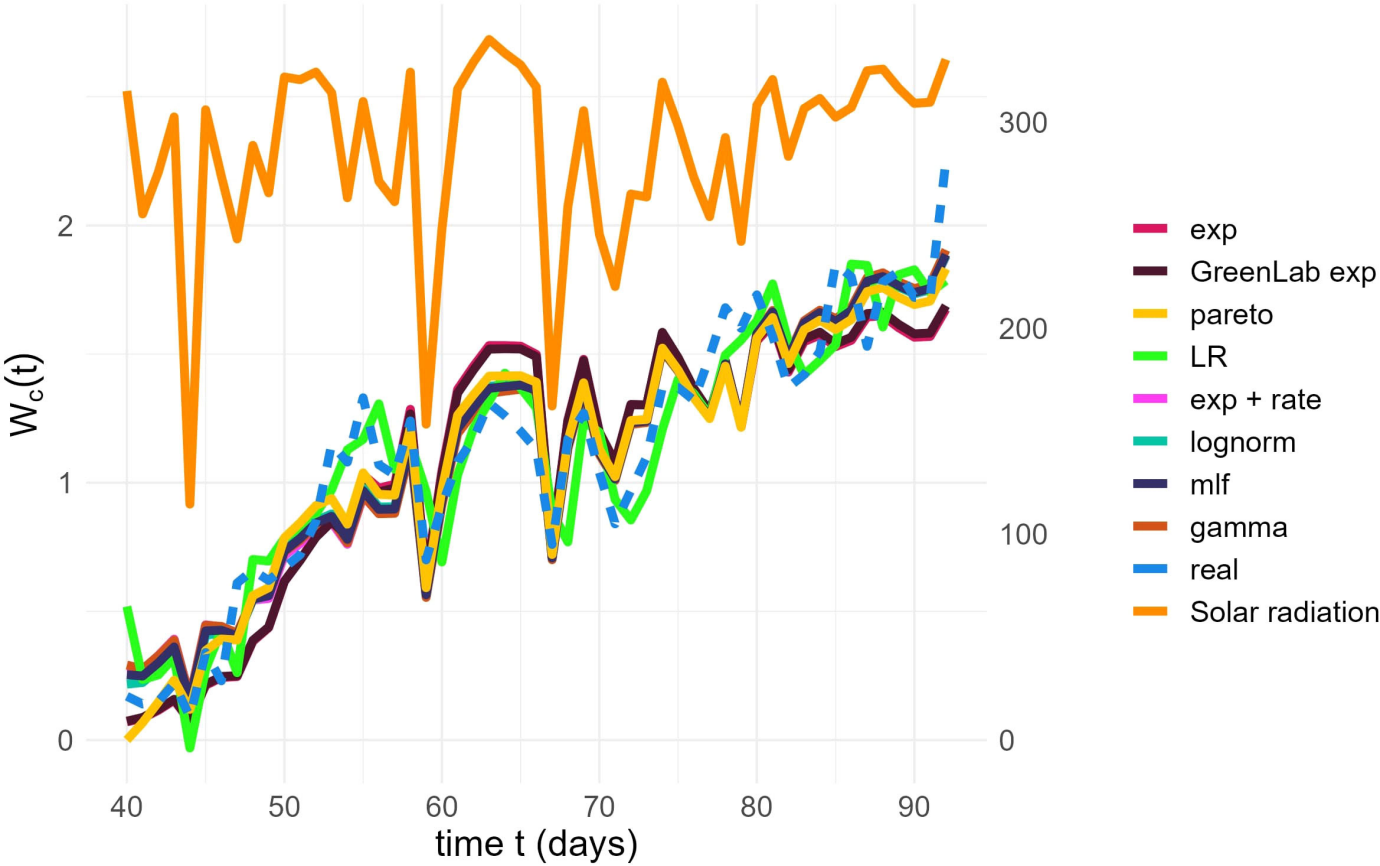
Final fit of the models (solid lines) on the real data (dashed line). Time (days), represented on the x-axis, runs over the days of observation, with *t* = 1 being the day the seed was planted. The left y-axis represents the Water Consumption at time *t*, in liters. The right y-axis represents values of Avg Solar Radiation (*W/m* ^2^). The evolution of Solar Radiation is plotted at the top of the graph, with a dark orange color.

Observing the temporal change of the estimated LIR with the different methods described in Section 2.6.2 reveals that the pairs (*lognormal*, *GreenLab-exp*), and (*gamma*, *lognormal + rate*) exhibit similar trends (**Figure 2**). This similarity is even more visible when the LIR is normalized by its maximal value and displayed with respect to *SW_c_*, as shown in the **Supplementary Material (Appendix 4)**. As the optimization procedure revealed, there is a compensation effect between *θ*_1_ and the *LIR* scaling, thus justifying the normalized representation in **Appendix 1.3**. However, the *pareto* and *mlf* methodologies demonstrate distinct trends that can be clearly differentiated from the others. The unique trend of the *pareto* methodology is also evident in **Figure 3**, where it manages to track the initial and final trends concurrently during the observation period, as opposed to the other methods, which are only capable of capturing either the beginning or the end trend, but not both simultaneously. Another notable result concerns the grouping of the best-performing models according to the BIC criterion (**Table 5**). The estimated LIR resulting from the best representative of these models is also shown in **Figure 2**.

### 3.3 Prediction results

The results of the predictive analysis revealed that the *pareto* and the LR models exhibited the best predictive performance both under known and unknown but predicted Solar Radiation, indicating their relative superiority within the context of our investigation (**Table 6**). However, the *lognormal* and the *mlf* models were slightly inferior and almost equivalent between them in both settings of Solar Radiation, followed by the gamma model. Surprisingly, it is crucial to acknowledge the underperformance of the *lognormal, GreenLab exp* and *lognormal + rate* models, which implement a methodology similar to the Beer-Lambert law. Compared to other models, these models’ inferior performance underlines the importance of generalizing the BL-law for optimizing performance.

**Table 6:** Prediction summary among the different suggested methods discussed in (6) and in (12), under: (left) Solar Radiation assumed to be known (right) an additive normal *N*(0,20^2^) noisy prediction setting.

	Version	RMSPE	RMSPE with noise
1	pareto	**0.194**	**0.202**
2	LR	0.205	0.212
3	mlf	0.226	0.234
4	lognorm	0.217	0.237
5	gamma	0.234	0.254
6	GreenLab exp	0.282	0.29
7	exp	0.296	0.319
8	exp + rate	0.341	0.364

Methods are compared using the RMSPE. The ordering is performed under the setting with noise. Bold lines indicate the "best" values according to the criterion under consideration.

## 4 Discussion

### 4.1 Different possible uses of crop models for predicting water consumption without plant information

Our primary objective was to investigate methodologies for modeling and predicting Water Consumption in tomato plants by utilising concepts derived from the crop models but without any information on the crop. While ambitious, this objective is grounded in uncovering hidden patterns within the crop’s behavior through the model’s learning process, particularly patterns of light interception. This approach is inherently interdisciplinary, combining methodologies from data science, statistics, and biology to address a complex biological problem.

Initially, we focused on the well-researched GreenLab model [Yan et al. (2004); De Reffye et al. (2021)], but the methodology could be considered generic and applied to other FSPMs (Sievänen et al., 2014) or crop models of tomato [e.g. Marcelis et al. (2008)]. Various strategies for using a mechanistic model in such a setting can be considered, each with a unique potential role. The first strategy revolved around employing GreenLab as a completely pre-fitted model, as done in Chew et al. (2014) for the case of *Arabidopsis*. However, the issues here concerned uncertainties and discrepancies among various genotypes. The second strategy would be to estimate all the parameters of GreenLab, but due to the incomplete dataset and the current experimental protocol, this strategy proved infeasible as the model exhibited identifiability problems, similar to the ones reported in Letort et al. (2012) for *Coffea* trees when only compartment data are available. Such an estimation procedure would necessitate destructive plant data, at different growth stages, for estimating the sink strengths and their variations Guo et al. (2006). The third strategy involved using GreenLab as a partially pre-fitted model, estimating only a fraction of its parameters. This was done for the *GreenLab exp* model, where only two parameters of the production equation were estimated. This strategy also encompassed the use of GreenLab as a submodel, assuming a similar pattern for the globally allocated biomass fraction to the leaves, as done for the *GreenLab rate*, or by combining some of its basic principles in the proposed SSiE models (e.g. proportionality between biomass production and water consumption is retained as an underlying hypothesis).

### 4.2 Summary of our main findings

The proposed models, employing *pareto*, *LR*, *mlf* or *Lognorm*, yielded comparable predictive outcomes

(RMSPE 0.2-0.23). In the context of our problem formulation, which involves one measurement of Water Consumption per day and relies solely on climatic data, this RMSPE translates to an error of approximately 215ml per day. This level of accuracy can contribute to the sustainability of agricultural practices by optimizing water usage. Importantly, the *pareto* and *mlf* models are feasible for application in a scheme of one measurement per day. However, both of them have disadvantages. The pareto model presents some identifiability issues among the *µ*_0_ and *θ* parameters, which warrants further investigation. On the other hand, the *mlf* is computationally heavy, a disadvantage that can be minimal in a scenario with only one measurement and only one day to predict. Despite these challenges, the models remain viable choices for real-world applications. Even though *lognormal + rate* and *gamma* models do not present equivalent results as the aforementioned, the LIR estimated by these methods, approximately 80%, are similar to the results reported in Wilson et al. (1992) and Ohashi et al. (2022). Measurements at 7 farms showed that in the summer season, the light interception was on average 90%, with values varying between 86% and 96%” in Heuvelink et al. (2004), with reported densities of 2.3 and 3.4 stems per *m* ^2^, in contrast to our case, where the reported density is 5 stems per *m* ^2^.

This work can be considered as a methodological proposition for determining the LIR profile with only a subset of the variables routinely measured by professional growers in a hydroponic setting, i.e., Water deficit volume, Solar Radiation. Interestingly, the profiles of LIR that we obtain in **Figure 2** are consistent with those reported in the literature [(Duursma et al., 2012), Ohashi et al. (2022)]. Selecting the models with the best predictive performances seems a reasonable strategy. Nonetheless, this approach warrants further empirical validation. Future research could focus on quantifying the diffusion of light in relation to distinct plant attributes and may include virtual experiments [as in (Duursma et al., 2012)].

### 4.3 Modeling light interception and its relation with plant growth

The amount of energy a crop captures, crucial for modeling crop growth and yield, is largely determined by canopy light interception (Higashide and Heuvelink, 2009). There is, however, currently no consensus on how light interception should be modeled: Liu et al. (2021) reviewed the canopy light interception models of 26 crop models of wheat and reported that the uncertainty in simulated wheat growth and final grain yield due to the different light models could be as high as 45%. The light interception modules form a continuum of approaches that range from simple (empirically or theoretically grounded) relationships between some characteristics of the photosynthetically active parts of the plant [usually LAI, e.g., Sarlikioti et al. (2011)] and the way they intercept light, to complex scene illumination algorithm, incorporating a precise 3D geometric representation of the plant [e.g. in Schipper et al. (2023)]. Our work contributes to that line of research, proposing a new cost-effective methodology to assess the time course of the LIR through its dependence on the water consumption profile. Our results further address the discussion of the need for generalizations or alternatives to Beer-Lambert law. Shabanov and Gastellu-Etchegorry (2018) and Casasanta and Garra (2018) have already proposed theoretical suggestions in this direction, and we believe our work presents a practical application of these theories.

Various variables have been employed in the literature to characterize light interception [e.g. STAR (light interception per leaf area) in Oker-Blom and Smolander (1988); Duursma et al. (2012), FIPAR (Fraction of PAR intercepted by the photosynthetically active radiation elements of canopies) in Liu et al. (2021)]. In this context, we utilize the Light Interception Ratio (LIR) that characterizes light interception per soil unit, a term we have intentionally left loosely defined. In our usage, this is primarily because LIR is a more empirically determined global variable rather than one rigorously derived from mechanistic principles. Nevertheless, we anticipate that it may still offer some interpretive value within the scope of our study. Our models cannot disentangle the different factors influencing light interception (leaf density, orientation, etc.) but provide a global representation of light interception at the plant scale, which is easy to obtain using routine measurements, and can assist in simple predictions of Water Consumption.

### 4.4 Limitations of the work

Our work presents important limitations that must be acknowledged. First, our modeling approach relies on strong physiological simplifications: e.g. neglecting soil evaporation and respiration of existing organs, constant radiation use efficiency, constant SLA, no influence of external environmental conditions except radiation and applied water volume, proportionality between light intercepted and photosynthesis [a more refined model here would have been to consider Farquhar’s photosynthesis model, for instance Farquhar et al. (1980)], proportionality between water consumption and biomass production. Regarding this last assumption, the ratio of biomass to transpiration [Water Use Efficiency (WUE)] is known to vary with weather, genotypes, and practices [Blankenagel et al. (2018); Bhaskara et al. (2022)]. Therefore, using a constant value is likely to be valid only in a limited range of environmental conditions that would have to be determined using a more extensive experimental dataset (Lanoue et al., 2017)

All these simplifications were required with respect to our objectives and our context of using only routinely recorded variables. They can, however, be considered applicable when describing the average growth of plants in standard conditions, and most of them are also laid in other models [Ma et al. (2022); Winn et al. (2023)].

An additional underlying assumption that deserves to be highlighted is that the *g* function is time-independent. In reality, *g* aggregates the effects of blade spatial arrangement, which determines the probability of a radiation ray being intercepted, the fraction of biomass allocated to the blades and the senescence of the leaves. This fraction decreases with time, especially due to the progressive appearance of fruits, whose demand competes with that of blades, a phenomenon that our SSiE models do not account for. However, in our case, because the time of observation is at a very later stage than the initial planting, this fraction is, in fact, nearly constant, taking values in the range (0.21-0.24), as simulated using GreenLab (Zhang et al., 2009). This explains why the models *exp* and *GreenLab exp* behave similarly.

In conclusion, we must acknowledge the limitations of our data, which prevent us from drawing strong conclusions from our results. Measuring and estimating the mean value of water consumption among only three plants could potentially introduce some errors because of the variance within them. Solar Radiation is measured outside of the greenhouse, which introduces the need for simulating an unknown transmission coefficient through the greenhouse: such coefficient is accounted for in the constant *θ*_1_ in model 12. Lastly, since we do not have access to light distribution measurements in our study, we cannot definitively conclude on the validity of our models by comparing our simulation outputs to real measurements, nor can we assess the stability of the values of the parameters of our models for different environmental conditions. Nevertheless, we believe that our work can be considered as a proof-of-concept for our proposed methodology and that the SSiE model appears promising for modeling Water Consumption.

### 4.5 Perspectives

In light of this, our future research will aim to apply further and investigate the utility of the SSiE model in predicting such quantities. The choice of distribution might be crop-dependent, and we aim to explore this idea in the future by acquiring data that would enable testing our models’ assumptions regarding the relationship between water consumption, crop architecture, and the different profiles of light distribution within the canopy.

From a methodological point of view, the current formulation is particularly adapted for Bayesian methods, which will allow for an easy way to quantify uncertainty and use the Bayesian predictive distribution for forecasting purposes. An online Bayesian method with sequential Monte-Carlo may be particularly relevant, and MCMC methods could also be applied for more efficient estimation, as in Logothetis et al., 2022. The comparison of MCMC with sequential Monte-Carlo for MLE was done in Trevezas et al. (2014).

## 5 Conclusion

In this study, we aimed to better understand plant water consumption, a subject of considerable importance for greenhouse management. The widely-used GreenLab model was not identifiable in our setting, even after compartmental simplifications, but it could be considered in other applications if at least partial information on the plant could be collected. Using similar physiological assumptions but in a probabilistic framework, we introduced the SSiE model as an alternative, directly applicable to water consumption, thus avoiding the need for biomass production as an intermediary variable. Despite the limitations of our data, the SSiE model provided some useful preliminary insights, particularly in the area of light interception over time. While these findings are still at a mostly theoretical stage, our proof-of-concept on our experimental dataset hints at the SSiE model’s potential utility for water consumption and light interception analyses.

The practical implications of these initial findings could be noteworthy and extend toward other crops and settings, offering a pathway to more efficient water usage in greenhouses.

## Data availability statement

The raw data supporting the conclusions of this article will be made available by the authors, without undue reservation.

## Author contributions

KF: Conceptualization, Data curation, Formal analysis, Investigation, Methodology, Project administration, Software, Visualization, Writing – original draft, Writing – review & editing, Resources. ST: Methodology, Project administration, Supervision, Writing – review & editing, Validation. VL: Methodology, Project administration, Supervision, Validation, Writing – review & editing.

## Supplementary material

Supplementary Figure 1 Normalized LIR w.r.t. cumulated water uptake.

## 1 Appendix

### 1.1 Appendix of GreenLab model for tomato

The integration of photosynthetic production is calculated using the Beer-Lambert (BL) law (Monteith, 1977), which is analogous to the approach employed in most process-based models:

(21)
$$Q\left(t\right)=E\left(t\right)\cdot \text{RUE}\cdot S_{p}\cdot \left(1-\text{exp }\left\{-k\;S_{L}\left(t\right)/S_{p}\right\}\right),$$

where during CD(*t*), *Q*(*t*) corresponds to the newly synthesized (dried) biomass, *E*(*t*) to the system’s energetic contribution (in our case, solar radiation), and RUE is the Light Use Efficiency (LUE) (the vegetation efficiency of converting radiative energy into biochemical energy through photosynthesis). Moreover, *S_p_* represents the projected surface potentially occupied by a single plant, while *S_L_* (*t*) stands for the plant’s photosynthetically active leaf area. This is calculated as the sum of the total photosynthetically active biomass of the blades multiplied by the specific leaf area (*SLA*). The variable *k* corresponds to the extinction coefficient in the Beer-Lambert law, and it is set to 0.8 for the tomato crop, as per Zhang et al. (2009). *Q*(0) = *Q*_0_ is the initial biomass of the seed. In our case, because of the difference in planting and first observation, the initial biomass will also be a parameter under estimation.

#### 1.1.1 Dry matter allocation

The biomass ascribed to every organ, spread from the common pool, is set proportional to its sink strength [Yan et al. (2004); De Reffye et al. (2021)]. In the context of mechanistic models for simulating dry matter partitioning, a common assimilate pool refers to a shared pool of assimilates from which various sink organs of a plant derive their growth resources. This implies that the plant does not segregate into distinct source-sink units, and thus, any resistance encountered during the transport of assimilates from source to sink would not influence the distribution of dry matter (Heuvelink, 1995). Sink strength adjusts during the period of organ expansion, following the same form of sink function for all organs of the same type *o* ∈ {*b,p,e,f*} in a cohort. A cohort is a set of organs of the same nature, created at the same CD by the parallel functioning of meristems.

If *T_o_* stands for the expansion duration of an organ of type (*o*) and *t* stands for its chronological age (days or CDs), then the sink strength is modeled by the function:

(22)
$$P_{o}\left(t\right)=p_{o}\cdot f_{o}\left(\frac{t}{T_{o}}\right),0\leq t\leq T_{o},$$

where *p_o_* is its relative sink strength (with respect to the blade’s one), *f_o_* (·) is the variation function of the sink related to its development, chosen as a Beta density function.

The sum of the sink strength of all organs is the Demand *D*(*t*) at a given time *t*:

(23)
$$D\left(t\right)=\sum_{o}\sum_{u=1}^{t}N_{o}\left(t-u+1\right)P_{o}\left(u\right),$$

where *N_o_* (*t* − *u* + 1) is the total number of organs of type *o* at time *t* that appeared at time *u* and are, therefore, of age *t* − *u* + 1. The biomass growth of an organ *o* varies on the value of its sink and the ratio supply produced to the previous cycle *Q*(*t* − 1) (2) by the current demand *D*(*t*) (23). The growth of an organ of type *o* that manifests during cycle *u*, while the plant is in a subsequent cycle *t > u*, can be articulated as:

(24)
$$\text{Δ}q_{o}\left(u,t\right)=P_{o}\left(t-u+1\right)\frac{Q\left(t-1\right)}{D\left(t\right)},$$

and the weight of the organ that appeared in cycle u when the plant is at age t is then:

(25)
$$q_{o}\left(u,t\right)=\sum_{j=u}^{t}\text{Δ}q_{o}\left(u,j\right).$$

#### 1.1.2 Beta sink function

In the initial GreenLab model (Yan et al., 2004), the sink function is defined according to a discretized beta law function:

(26)
$$f_{o}\left(\frac{t}{T_{o}}\right)=\frac{1}{M}{\left(\frac{t}{T_{o}}\right)}^{a_{o}-1}{\left(1-\frac{t}{T_{o}}\right)}^{b_{o}-1},0\leq t\leq T_{o},$$

where the parameters *a_o_* and *b_o_*, verifying the constraint *a_o_,b_o_* ≥ 1, drive the curve shape and *M* is a normalization constant usually modeled as the sum or the max over 1 ≤ *t* ≤ *T_o_*.

In this case, the parameters to estimate are:

(27)
$$\theta =\left(a_{b},b_{b},p_{p},a_{p},b_{p},p_{e},a_{e},b_{e},p_{f},a_{f},b_{f},S_{p},\text{RUE},SLA,Q_{0}\right)$$

The absence of *p_b_* arises from the standard practice of establishing *p_b_* = 1 as a reference point [Dong et al. (2008); Zhang et al. (2009)].

A dimensionality reduction approach, discussed in (Dong et al., 2008), stabilizes the sum of the two parameters *a_o_, b_o_*. The value of 5 has been specifically chosen for tomato plants, as it has been observed to produce fine results. Under this assumption, the parameter’s space dimension is reduced by 4.

### 1.2 Probabilistic justification of the SSiE model

Let us now try to justify the rationale behind the discussion in Section 2.6. The radiative input *E*(*t*) could be mapped to the interval [0*,E*(*t*)] representing an uncountable number of points potentially available for biomass production. At each point *u* of the interval, one could attach a Bernoulli random variable, say *X_u_* (*t*), deciding whether the point *u* will enter the system or not. If it enters the system, then it brings an infinitesimal contribution to biomass production; otherwise, it is rejected and exits the system. One could still keep the independence assumption and assume that there is a common probability *p*(*t*) of the radiative points entering the system, but there is a price to pay. If we assume that the radiative input is a realization of the stochastic process ${\left\{Z_{u}(t)\right\}}_{u=0}^{E(t)}$ , where the sample (observed) paths would be an interval of points consisting of 0^′^ *s* and 1^′^ *s*, then it can be proved with tools from probability theory that the resulting processes are not measurable.

To give an interpretation of this nonmeasurability concept, it roughly corresponds to the idea that it would be impossible to associate the usual notion of length to the set of points that entered the system and the set of points that exited the system in this ideally conceptualized experiment. Luckily enough, there is still a solution, and it gives a formal justification for our intuitive approximations. It resides in the disintegration theorem (Chang and Pollard, 1997), a result of measure and probability theory. In fact, this theorem gives very powerful tools and a more intuitive approach to the definition of conditional probability and conditional expectation than the one that is usually presented in standard probability textbooks. A formal description of this theorem and related conditions for its validity would be out of the scope of this paper, and we refer to Chang and Pollard (1997). However, we describe the basic ingredients and the result we need in our context.

Instead of selecting points from the interval [0*,E*(*t*)], one could think that the same interval is actually a bundle of Bernoulli experiments, where each one of them is realized when the point *u* is “activated”. Formally, one needs a measure space which consists of the set *Y_t_* := [0*,E*(*t*)] × {0,1}, an appropriate measure *µ* and a function *π* : *Y_t_* → [0*,E*(*t*)] (usually the projection function) which disintegrates the measure *µ* into a family of measures ${\left\{{µ}_{u}\right\}}_{u=0}^{E(t)}$ , such that for a measurable *A*

(28)
$$\mu_{u}(A)=\mu_{u}\left(A\cap \left\{\left(u,0),(u,1\right)\right\}\right)$$

and induces the measure *ν* = *µ* ° *π* ^−1^ on [0*,E*(*t*)]. In our case, the choices are rather simple. Each *µ_u_* is “living” (has its support) on the fiber {*u*} × {0,1} and behaves as a Bernoulli measure, while the induced measure *ν* should be the Lebesgue measure restricted on [0*,E*(*t*)]. In this way, the disintegration theorem justifies the following way of computing the measure of a measurable set *A*:

(29)
$$\mu \left(A\right)=\int_{0}^{E\left(t\right)}\mu_{u}\left(A\right)\;du,$$

where *µ_u_* (*A*) is given by (28), and the integral should be understood in the Lebesgue sense. We are now ready to make the correspondence with the computation of the totally produced biomass at time *t*. Since the set *B* = [0*,E*(*t*)] × {1} corresponds to the set of all active points, in order to assess the totally absorbed radiative input, we just have to compute

(30)
$$\mu \left(B\right)=\int_{0}^{E\left(t\right)}\mu_{u}\left(B\right)\;du=E\left(t\right)p\left(t\right),$$

since *B* ∩ {(*u*,0),(*u*,1)} = {(*u*,1)} and *µ_u_* ({(*u*,1)}) = P(*A_u_* (*t*)) = *p*(*t*). Multiplying by RUE·*S_p_* to transform into biomass, we get the expected approximation result given by (9). It is also interesting to notice that the constant probability *p*(*t*) is actually playing the role of a constant flow (with respect to the incoming radiation) of biomass production. The discussion is continued in paragraph 2.6.1.
