# Supplementary figures and images for: Predicting tomato water consumption in a hydroponic greenhouse: contribution of light interception models
Source: Front Plant Sci. 2023 Nov 28;14:1264915. doi: 10.3389/fpls.2023.1264915 (PMC10714001; doi:10.3389/fpls.2023.1264915)

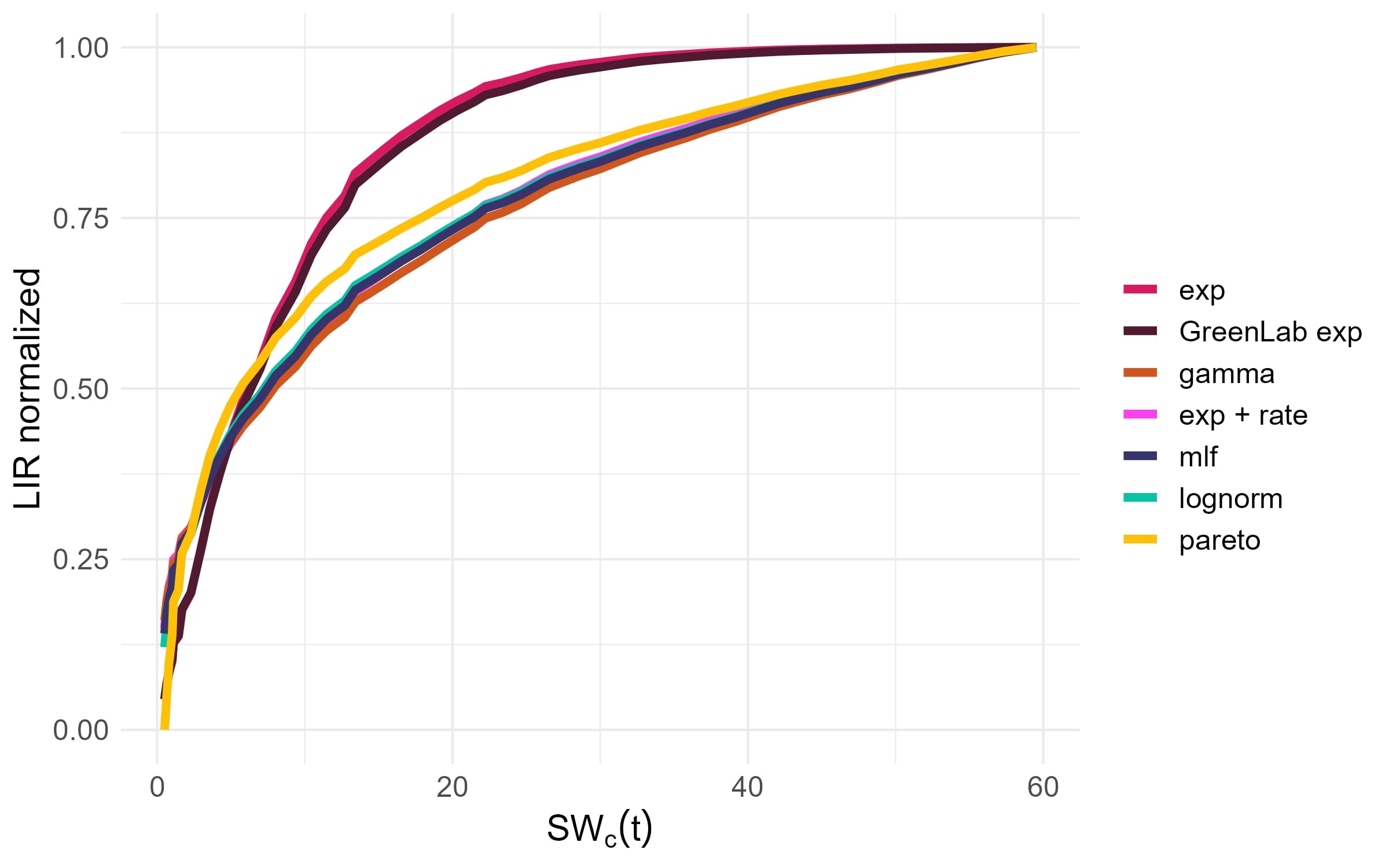

Supplement: Supplementary Figure 1 — Normalized LIR w.r.t. cumulated water uptake. [file Image_1.jpeg]
